# Relation of General Arteriosclerosis to Certain Ocular Conditions

**Published:** 1914-05

**Authors:** 


### Relation of General Arteriosclerosis to Certain Ocular Conditions.

Elsworth Smith (Annals of Ophthalmology, January, 1914) states that though arteriosclerosis is a general disease, the arterial circuit is not always uniformly involved. In some cases the brunt of the disease is spent on the heart, in others on the kidneys, the brain, digestive tract, etc., and thus according to the distribution of its ravages arise the various symptoms making up the complex picture of the disease. The end comes to its victims at times suddenly through an acute cardiac dilatation or an apoplexy, or more gradually with all the long drawn-out symptoms of an uncompensated chronic lesion or chronic uremia.

As to the prognosis in this malady we of course must realize that the organic changes in the vessel wall cannot be undone, any more than a damaged valve in the heart can be replaced by a normal one; but much can be done toward arresting and limiting the process by removal of the causative factors. The earlier, of course, the condition can be detected, the better the outlook, other things being equal, for a comfortable and useful life during a number of years; but the point I wish especially to emphasize is that the condition must be recognized early.

This brings us to the subject matter proper, viz., that the eye is the organ where arteriosclerosis is often earliest manifested, and where it can be most accurately appreciated through study of the eye grounds, and so impressed have the internists and surgeons been with the great value of the ophthalmoscope as a diagnostic aid in the affection under consideration, and in many other maladies, that ophthalmoscopic examinations are now practiced as a routine procedure.

Foremost, of course, among the ocular lesions of general arteriosclerosis ranks retinitis, or the so-called albuminuric retinitis. It is especially in the degenerative form of the so called albuminuric retinitis, rather than in the inflammatory variety, that arteriosclerosis is most typically manifested, presenting in the ophthalmoscopic picture the thickened, tortuous arteries, at times strapping of the veins, hemorrhages, exudations, degeneration, etc., all of which signs point to a degeneration of the retinal vessels with increased arterial tension, both direct results of the disease.

Aside from the various operative measures in the eye affections alluded to, the mainstay in the treatment appears to be bichlorid of mercury and iodid of potassium; not only in cases with a luetic element, but also those non-luetic. And while these are most valuable drugs, and doubtless do a great deal of good, yet I desire now to emphasize a second point, which is that all ocular conditions which can reasonably be assumed to be dependent upon general arteriosclerosis should be given the benefit of a general therapeutic regime along the following lines:

1. When the conditions in the eye are so grave as to threaten the integrity of the organ the patient should be immediately placed at complete rest in bed, with the hope of thereby assisting in the reduction of the arterial tension and thus averting a calamity in the eye as well as in the heart, brain, etc.

2. That if the condition in the eye or general condition be not urgent enough to require complete rest in bed, then the patient should be urged to pursue the even tenor of his way, free as possible from all sources of worry and strain. The strenuous life must be dropped, and while it is not well to enjoin an active man from all occupation, yet he must be taught moderation at work.

At the proper time exercise of a moderate, graduated kind must be insisted on, such as walking, golf, etc., and what is most important, a careful diet. Alcohol, tobacco and meat must at first be denied, and later, at least greatly limited for such patients. Milk, and especially buttermilk, must be taken freely, bringing to mind Metchnikoff's theory of the control of intestinal fermentation in the large intestine by lactic acid products. As to medicinal remedies, iodid of potassium has a double value because, experimentally, it has been stated arteriosclerosis may be prevented if coincidentally with adrenalin iodid of potassium is given. It is thought by some to have an influence in the lessening of arterial tension.

Of drugs having a more direct effect on lessening of blood tension, we have the nitrite group, as nitroglycerin 1 to 5 mm. of 1 per cent solution three or four times daily, but though given in large doses, its effect is quite transient. Nitrite of sodium in 1 to 4 grains, and erythol tetranitrate, one-half grain every hour to six hours, have a more permanent action.

All these remedies of the group of vasodilators are, of course, mainly indicated to meet certain emergencies where disaster is threatened by excessive hypertension. In the main, dependence must be had in the management of this class of cases on diet, rest, regulated exercise and careful and thorough elimination.

